# The Role of Right Inferior Parietal Cortex in Auditory Spatial Attention: A Repetitive Transcranial Magnetic Stimulation Study

**DOI:** 10.1371/journal.pone.0144221

**Published:** 2015-12-04

**Authors:** Debra S. Karhson, Jeffrey R. Mock, Edward J. Golob

**Affiliations:** 1 Program in Neuroscience, Tulane University, New Orleans, LA, United States of America; 2 Department of Psychology, Tulane University, New Orleans, LA, United States of America; 3 Center for Aging, Tulane University, New Orleans, LA, United States of America; University of British Columbia, CANADA

## Abstract

Behavioral studies support the concept of an auditory spatial attention gradient by demonstrating that attentional benefits progressively diminish as distance increases from an attended location. Damage to the right inferior parietal cortex can induce a rightward attention bias, which implicates this region in the construction of attention gradients. This study used event-related potentials (ERPs) to define attention-related gradients before and after repetitive transcranial magnetic stimulation (rTMS) to the right inferior parietal cortex. Subjects (n = 16) listened to noise bursts at five azimuth locations (left to right: -90°, -45°, 0° midline, +45°, +90°) and responded to stimuli at one target location (-90°, +90°, separate blocks). ERPs as a function of non-target location were examined before (baseline) and after 0.9 Hz rTMS. Results showed that ERP attention gradients were observed in three time windows (frontal 230–340, parietal 400–460, frontal 550–750 ms). Significant transient rTMS effects were seen in the first and third windows. The first window had a voltage decrease at the farthest location when attending to either the left or right side. The third window had on overall increase in positivity, but only when attending to the left side. These findings suggest that rTMS induced a small contraction in spatial attention gradients within the first time window. The asymmetric effect of attended location on gradients in the third time window may relate to neglect of the left hemispace after right parietal injury. Together, these results highlight the role of the right inferior parietal cortex in modulating frontal lobe attention network activity.

## Introduction

Attention is a limited resource whose allocation to locations, features, and objects is evident by enhanced detection and identification of stimuli [1,2]. Previous work shows that the spatial tuning of auditory attention can be organized in a gradient-like manner [3]. When a target location is specified with a cue reaction times are fastest at the cued location and progressively increase to stimuli presented at greater distances [4,5]. Data from these auditory studies are consistent with results from visual spatial attention experiments that have also defined attention gradients [6–9].

Although behavioral findings strongly support attention gradients in the auditory modality, only a few studies have examined their neural correlates. Teder Salejarvi et al (1998, 1999) found that two event-related potentials (ERPs), an attention-related negativity and the P3b, indexed attention gradients when sounds were presented rapidly over a relatively small range of locations (18°-54°). The amplitudes of the attention negativity and P3b were progressively smaller at greater angular distances from the attended location [10,11]. Under similar conditions Arnott and Alain (2002) identified a gradient for an attention-related negativity [12].

We note that a main function of the auditory system is to act as an early warning system by panoramically monitoring the acoustic environment for infrequent events [13]. This ecological consideration motivated a study by our lab where stimuli were presented at a wider range of locations and at a slower rate. Findings demonstrated that increases in angular distance between a distractor and the attended location were accompanied by larger amplitudes of the P3a component [14]. The P3a is a well-studied ERP component that is associated with attentional orienting [15]. As will be described in more detail in the “Data Analysis” section, the gradient for attentional orienting can be estimated by calculating the slope of how much P3a amplitude and other EEG measures change as a function of distance between the location of distractor stimuli and the current focus of attention. The slower stimulus rate allowed for the development of late frontal slow waves, which began at ~500 ms contralateral to the stimulus’ location, lasted for at least 1 sec, and were most negative for distractors closest to the attended location.

Patient data provide important clues about which neuroanatomical areas are vital for normal spatial attention. In particular, hemineglect patients have graded attention deficits that are typically worse for the left side of space, and are evident in visual [16–19], somatosensory [20,21], and auditory modalities [22–24]. Progressive improvements in attention are observed moving from the contralesional to ipsilesional hemifield in visual attention [25–27]. Note that graded spatial attention deficits are only one prominent symptom of hemineglect. Others include impairments in the ability to sustain attention or respond to salient stimuli regardless of spatial location [28]. Hemineglect is most common after injury to the right inferior parietal lobe, but is also found after injury to the right prefrontal cortex, right insula, and the basal ganglia [29].

Although compelling, the interpretation of lesion data is limited due to the potential development of atypical functioning of spared regions and damage to fibers of passage [30]. The use of repetitive transcranial magnetic stimulation (rTMS) in healthy subjects can provide convergent evidence regarding structure-function relationships. Relatively low frequency rTMS (≤1Hz) can transiently interfere with information processing within the area receiving stimulation [31–35]. In addition, rTMS affects activity in distant regions that are densely connected to the site of stimulation, suggesting a widespread influence of rTMS within neural networks [36]. Previous work has also shown that TMS to the superior temporal as well as occipital cortex can influence spatial hearing [37].

The main purpose of this study was to determine if the right inferior parietal cortex contributes to the patterns of ERP responses previously associated with attentional gradients [3,14]. Our strategy was to first use ERPs to define neurophysiological correlates of presumed attention gradients. This was done by having participants attend to a target location on the far left or right (counterbalanced across blocks), and presenting non-target distractor stimuli spaced at 45° intervals away from the target location. As in our previous study [3], gradients were defined by examining ERP amplitudes as a function of angular distance from the target. The rationale is that if sustained attention to a target location establishes a diffuse attention gradient then there should be systematic changes in EEG measures to a stimulus when it is presented at different locations relative to the currently attended location. In essence, the non-target distractors serve as a way to probe for neurophysiological indicators of the gradient due to differences in how the same stimulus is processed when it is closer vs. farther from the target.

The ERP gradients were measured before and after low frequency rTMS was applied to the right inferior parietal cortex. Previous work has shown that rTMS can elicit mild attentional deficits in controls similar to those observed in hemineglect patients [38–41]. The main hypotheses were informed by the neglect and neurophysiological literatures outlined above. The first hypothesis was that if rTMS induces a contraction of attentional gradients then a reduction in activity at the farthest locations from the target was expected. The second hypothesis was that application of rTMS to the right inferior parietal would lead to a decreased slope of ERP amplitudes as a function of distance from the attended location. Finally, the third hypothesis was that rTMS would induce a general modulation of processing of non-target distractor stimuli, particularly when attending to the left as seen in hemineglect patients. As a preview of the results below, the findings supported the first and third hypotheses.

## Methods

### Participants

This study was approved by the Tulane University Institutional Review Board, and was consistent with the Declaration of Helsinki. Written informed consent was obtained from all participants. Tulane University graduate students (n = 16, age: 24.9 ± 3.5 yrs., M/F: 8/8, 14/16 right handed) completed a modified auditory oddball task as electroencephalography (EEG) was recorded. Audiometric testing verified that all participants had pure tone thresholds within the normal range (500–8,000 Hz; Maico, Eden Prairie MN).

### Design

**Fig 1:**
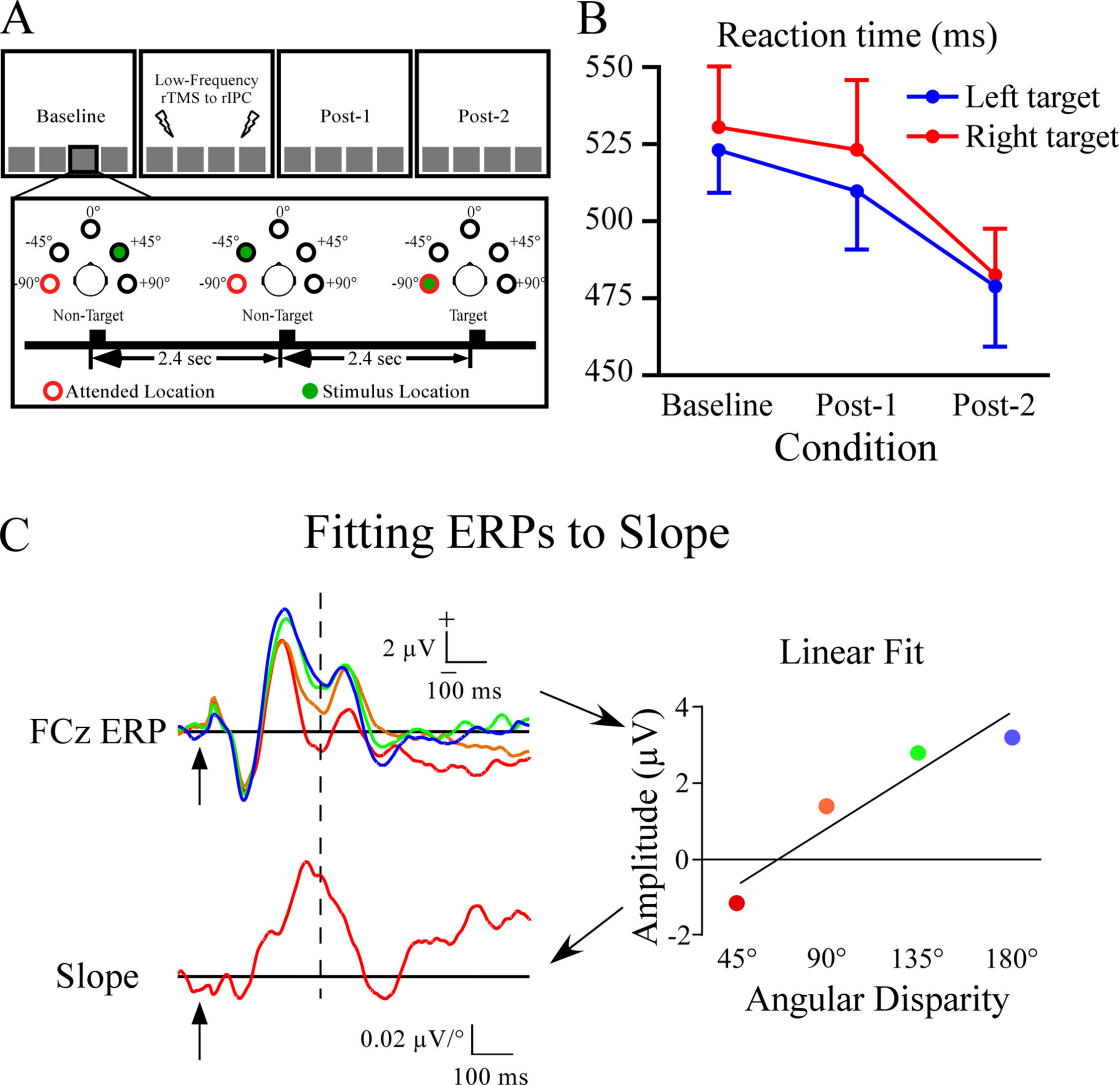
Experimental design and behavioral results from spatial target detection task. A) In this excerpt from a sequence the target location is at the left side (-90°). The four conditions are shown at the top, with the Baseline, Post-1 and Post-2 examined in this report. B) Plot of reaction time for left and right targets across the three conditions. There was a progressive reduction in reaction time across conditions, and did not differ among left or right target locations. C) Illustration of the method to calculate the slope of ERP amplitudes across the four non-targets at each sample point (“FCz ERP”). Data showing a linear fit across the non-target ERP amplitudes at 300 ms latency are shown at the right (labeled “linear fit”). The slopes at each sample point from 0–1000 ms were calculated and plotted as a waveform of the slope values (uV/degree) as a function of time (bottom, labeled “slope”). The vertical line in 1C indicates the time point for the amplitudes that are plotted in the “linear fit” panel.

The experiment had four conditions: Baseline, rTMS, and two conditions that followed the rTMS, termed Post-1 and Post-2 (Fig 1). After baseline participants were given 0.9 Hz rTMS to the right inferior parietal cortex for 16 min (~864 pulses) while performing two blocks of the auditory attention task. The main analyses compared baseline to two post-rTMS conditions to assess the impact of rTMS on attention-related ERP components. Analysis of the rTMS block is not discussed in this paper. Each condition had four blocks of trials. In two of the blocks participants attended to a far left (-90°) target location. In the other two blocks the target location was at the far right (+90°). Blocks of left and right target locations were randomized and counterbalanced across participants, and EEG was recorded in all conditions. The goal was to examine the formation of attentional gradients at non-target locations. Consequently, we focused the ERP analysis on non-target distractors in the baseline and post-rTMS conditions. The analyses below included efforts to distinguish transient rTMS effects on ERPs from changes in ERPs that could be due to the effects of repeated testing. Transient effects of rTMS were evident when ERP measures in the post-rTMS 1 block differed from the earlier baseline and later post-rTMS 2 blocks. When present, this “v-shaped” or upside down “v-shaped response” across trial blocks provides evidence against non-specific rTMS effects as well as ERP changes due to the passage of time, repeated stimulus presentations, and practice. Any result that was found in one attention location but not the other also provided an internal control against non-specific effects.

### Behavioral task

In each trial block participants listened to a random sequence of sounds from five possible locations that were evenly spaced between -90° and +90° (Fig 1). Participants were instructed to attend to a lateral target location on the left (-90°) or right (+90°), and to respond with a button press with dominant hand when a stimulus was presented at the target location. Interspersed among target stimulus presentations, non-target sounds were presented at four angular distances relative to the target location (non-target locations). Probability of sound presentation was 0.20 for each location, with an interstimulus interval of 2.4s. Prior to the start of each block the investigator notified participant of the target location (either -90° or +90°) and to respond rapidly while maintaining high accuracy.

### Stimuli

All stimuli were presented to participants via insert ear headphones. For detailed background on constructing virtual auditory stimuli see [42]. A single broadband white noise burst (0.1–10kHz, 200 ms duration, 5 ms rise/fall time, ~60 dB nHL, 40.1 kHz sample rate) was processed to generate realistic perceptions of sounds originating from different locations relative to the participant’s head. The same white noise sample was processed to add natural cues used to define the location of a sound, specifically interaural time and level differences as well as head related transfer functions (Tucker-Davis Technologies, Gainesville, Florida, USA, System II and the University of Wisconsin). The five sounds were constructed to correspond to five locations in the frontal horizontal plane: left (-90°, -45°), midline (0°), right (+45°, +90°). Participants were pre-tested to ensure that sounds were perceived in the intended locations by having subjects mark on paper the perceived location of each sound relative to the head. In the pre-test the perceived location of each of the five stimuli was separately assessed by repeatedly presenting the stimulus for a given location (750 ms inter-stimulus interval). While listening to the repeated stimulus each participant would mark the perceived location on a sheet of paper that had lines for the midline and interaural axes, which were connected by an arc to represent the frontal azimuth [43,44]. This was repeated until the participant had marked the perceived location of all five stimuli that were used in the experiment. While the 0° and ± 90° locations were always perceived accurately, some participants received additional practice to clearly designate the -45° and/or +45° locations.

### Electrophysiological recordings

Electroencephalography (EEG) was recorded in an electrically shielded, sound attenuating booth and recorded from a 64-channel system (Compumedics Neuroscan, Charlotte, NC) using an Ag/AgCl 64-electrode cap. Four electrodes measured vertical and horizontal EOG, and were placed above and below the left eye and lateral to each eye. Impedances were maintained at ≤ 10 kOhms and a reference electrode between Cz and CPz was used during data collection. EEG and EOG were digitized at 5,000Hz (the fast sampling rate was used to examine TMS-evoked potentials not presented here). Offline processing included downsampling to 500 Hz, re-referencing to an average reference configuration, and eye-blink artifact correction which was done using independent component analysis [45]. Data were epoched into 1,200ms sweeps (-200 to 1000ms relative to stimulus onset) and separately averaged according to target and non-target locations in the attend left and attend right blocks. Thus, for every target location (-90° or +90°) there were four non-target ERPs at locations that were 45°, 90°, 135°, and 180° away from the target location.

### Offline repetitive TMS

Repetitive TMS was applied using a standard 70-mm figure eight shaped coil connected to a magnetic stimulator (Magstim Rapid2, Whitland, Wales, UK). The coil was oriented in a posterior-anterior direction, and rTMS was delivered at 0.9 Hz, with intensity set at 75% of stimulator output for 16 minutes. As in previous studies, the location of the right inferior parietal cortex was estimated to be midway between electrodes P4 and P6 on a standard 64-channel 10–20 EEG system [38–40].

### Data Analysis

Behavioral responses to targets were analyzed for changes in reaction time and response accuracy (hits) when targets were delivered at the attended location. False alarms to non-targets were also recorded, and were not included in the EEG analysis. EEG potentials were band-pass filtered from 0.1–16 Hz (12 dB/octave).

**Fig 2:**
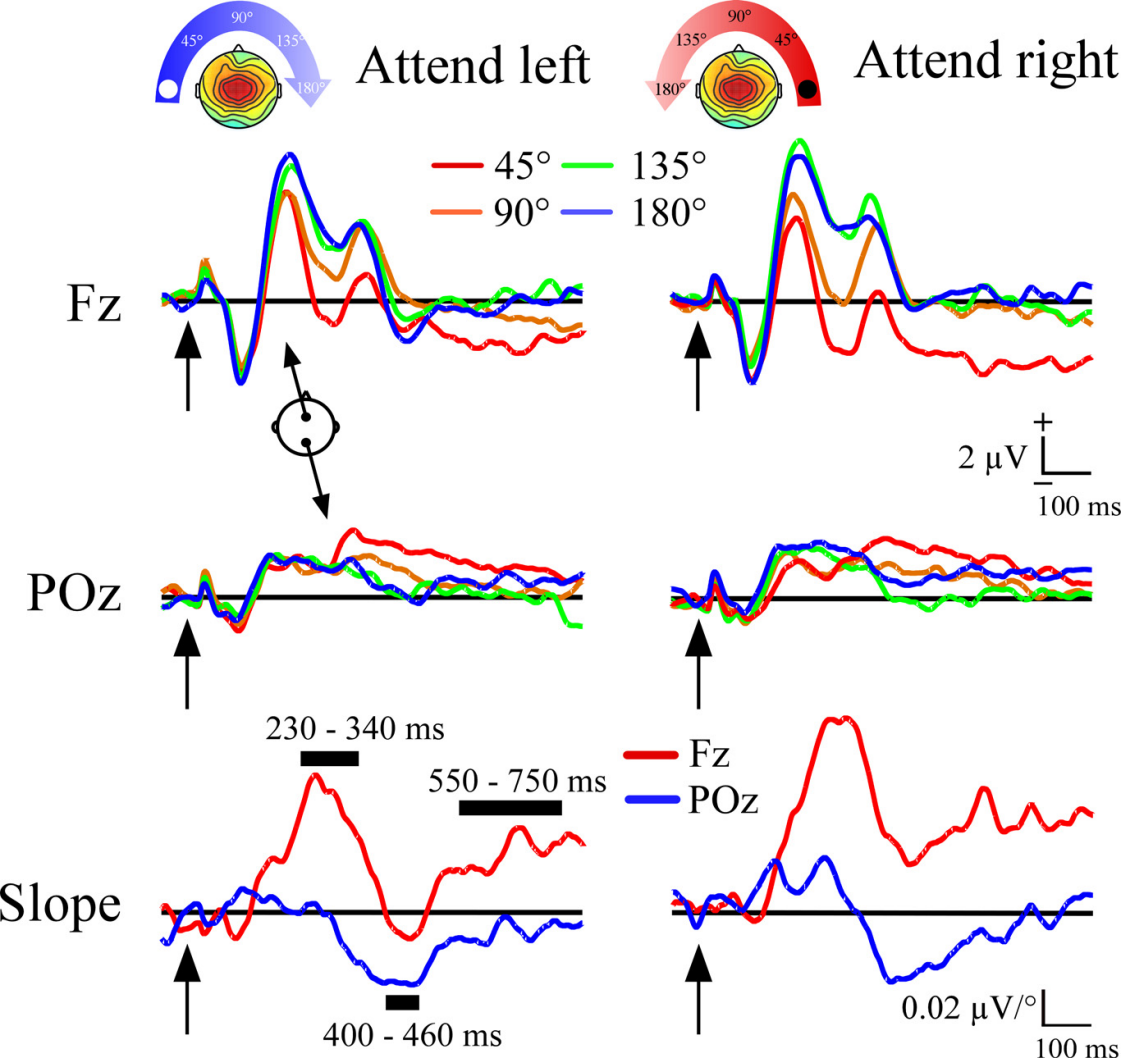
Event-related potentials to non-target distractors as a function of angular distance from target location. The top and middle rows illustrate the basic gradient effects in the ERPs. The black rectangles indicate the three time windows where voltage was measured. Within the two windows at Fz there were progressively more positive potentials as a function of distance from the target shown. At the POz site the opposite pattern was seen, non-target potentials became more negative with greater distance from the target. The selection of time windows was based on plots of the slope calculated across all four non-target locations at each 2 ms time point, and are shown in the bottom row.

As with a previous publication [3] we fit a linear function at each sample point and electrode site to the voltage across the four non-target locations (see Fig 1C). Thus, the slope would be positive if voltage to the non-target ERPs at a given latency increased with distance from the target location. If voltage showed linear reductions with distance, then the slope would be negative. The slopes of non-target ERP responses were defined with respect to the target location (45°, 90°, 135°, and 180° from target location). The slope measure served as a way to capture with one number the gradient of non-target ERP responses as a function of their angular distance from the target. As in Mock et al. (2015), the slope measure had three prominent peaks (Fig 2). The first peak was a positive slope that was largest at frontal sites, and was measured with a 230–340 ms window at the Fz site. The second peak had a negative slope, was largest at posterior sites, and was measured at POz between 400–460 ms. The third peak was also centered at frontal sites and had broad, prolonged positive slope, which was measured with a time window of 550–750 ms at Fz. The Fz site was used because the topography of activity in the 550–750 ms window was not strongly lateralized, unlike prior reports [3,14]. The slope served as a guide for placement of the three time windows, which as described next, were used to quantify ERP responses that were then entered into standard ANOVA tests.

### Statistical Analysis

Significance in all analyses was defined as p < .05. Behavioral analysis used ANOVA tests to examine target reaction times and hits, and false alarms to non-targets. Factors included condition (Baseline, Post-1, Post-2) and target location (-90°, +90°). Preliminary analyses used signal detection theory to examine hits and false alarms, but there were no significant effects for d’ or c measures. Analysis of ERP data also included a factor of the 4 non-target locations. Follow-up testing used planned linear and polynomial contrasts. Non-target location was coded relative to the target location (45°, 90°, 135°, 180° away from target) to allow for a direct comparison between the left and right target conditions. The Greenhouse-Geisser correction was used for violations of sphericity, but for clarity the original degrees of freedom were reported below.

## Results

### Behavior

Reaction time, accuracy, and false alarms to targets were analyzed with ANOVA tests using factors of target location (-90°, +90°) and condition (Baseline, Post-1, and Post-2). For reaction time there was a main effect of condition (F_(2,30)_ = 8.7, p < .01) due to faster reaction times as the experiment progressed (Fig 1C). There were no significant effects for hits (mean = 85.2 ± 0.8%) or false alarms (mean = 1.4 ± 0.1%).

### Event-related potentials and slope measures

The event-related potential waveforms in the three conditions (Baseline, Post-1, Post-2) for both attended locations are shown in Fig 2 at the Fz and POz sites. Plots of slope measures calculated across the four non-target locations at each time point are shown at the bottom, with the Fz and POz sites superimposed in each target location condition. Taken together, the results show progressive increases in voltage to non-targets relative to target location, which are reflected in two positive frontal slope peaks. Interposed between the two positive slope peaks is a negative parietal slope peak. The negative slope peak indexed progressive reductions in voltage with greater non-target distance, and was maximal at ~430 ms. These three slope peaks were quantified using mean voltage in time windows time windows centered on each peak: 230–340 ms, 400–460 ms, and 550–750 ms. The three time windows were measured in each of the four ERPs that were elicited by non-targets, with separate measures when the target was on the left vs. right side. These time windows were also used in a prior publication [3] with the exception that the earliest time window was somewhat earlier and lasted longer (270–330 ms vs. 230–340 ms used here).

### 230–340 ms time window

**Fig 3:**
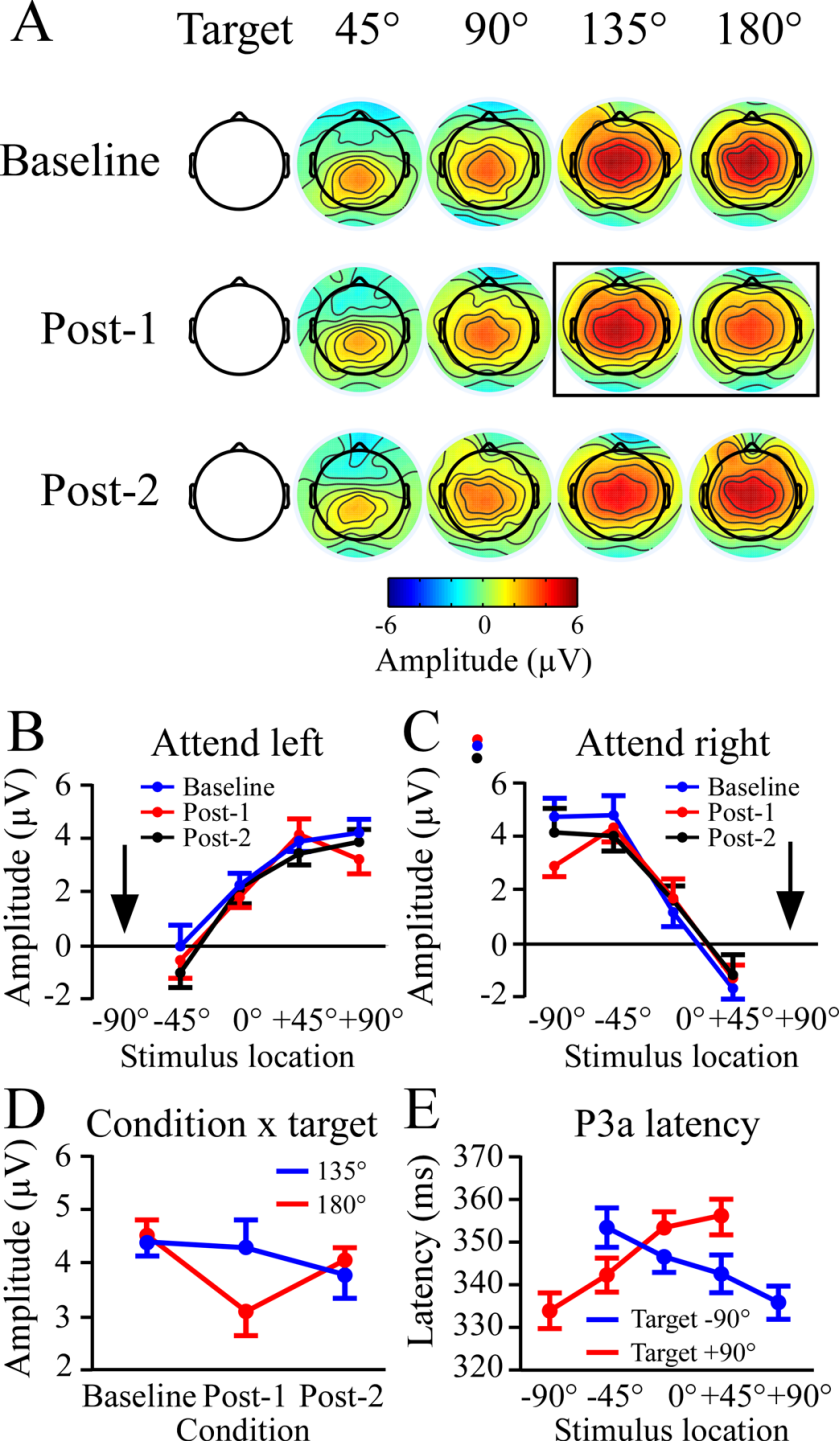
Non-target distractor potentials between 230–340 ms at the Fz site. This time window reflects the first peak of the non-target slope function shown in Fig 2, and corresponds to the P3a component in previous studies. A) Topographic plots of the 230–340 ms window across non-target locations in each of the three conditions. Results were collapsed across left and right target blocks because there was no difference between target locations. Box outline highlights the reduction in voltage between the 135° and 180° locations in the Post-1 condition. B, C) Voltage plots for non-target locations when attending to the left (B) and right (C). For both attended locations there were strong linear increases in voltage with greater non-target distance from the target. For B and C the arrow indicates the location of the attended target. D) Plot showing the significant reduction in 230–340 ms amplitude at the most distant non-target following rTMS. In the Post-2 condition the amplitude had recovered to baseline levels. E) P3a latency as a function of non-target location. Latencies were longest to non-targets near the target, and progressively decreased with more distance from the target location.

The main results from the 230–340 ms time window analysis are presented in Fig 3. The topographic plots in Fig 3A show progressive increases in frontal amplitude as a function of non-target location, but with a reduction at the far location only in the Post-1 condition. Results for the left and right targets were comparable, and collapsed together in the topographic plots. The window measures from electrode Fz were examined using a 3 (condition: baseline, post-1, post 2) x 2 (target location) x 4 (non-target location) ANOVA. There was an effect of non-target location (F_3,45_ = 61.5, p < .001), that was well-fit using linear trends (Target -90°: F_1,15_ = 71.5, p < .001; Target +90°: F_1,15_ = 157.5, p < .001)(Fig 3B and 3C).

The farthest non-target locations (135°, 180°) were of a priori interest because a decline in the edges of an attention gradient were expected if rTMS subtly mimics the spatial attention deficits in hemineglect. The topographic results suggest a transient frontal voltage reduction at the far location in Post-1, which is plotted in Fig 3D. This was examined in detail using paired comparisons between the 135° and 180° locations in each condition. Amplitudes among the two locations were nearly identical in the Baseline and Post 2 conditions, while in Post-1 amplitudes at 180° were significantly less than those at 135° (F_1,15_ = 6.9, p < .02).

The 230–340 ms time window coincided with portions of the P3a peak in previous studies [14,15]. Visual inspection showed that for both target locations the latency of the P3a peak decreased with greater non-target distance from the attended location (Fig 3E). To test this impression the P3a peak was measured and examined with a 3 (condition) x 2 (target location) x 4 (non-target location) ANOVA. There was a main effect of non-target location (F_3,45_ = 16.5, p < .001), which had significant linear fits for each target location (Target -90°: F_1,15_ = 13.0, p < .01; Target +90°: F_1,15_ = 30.1, p < .001).

### 400–460 ms time window

**Fig 4:**
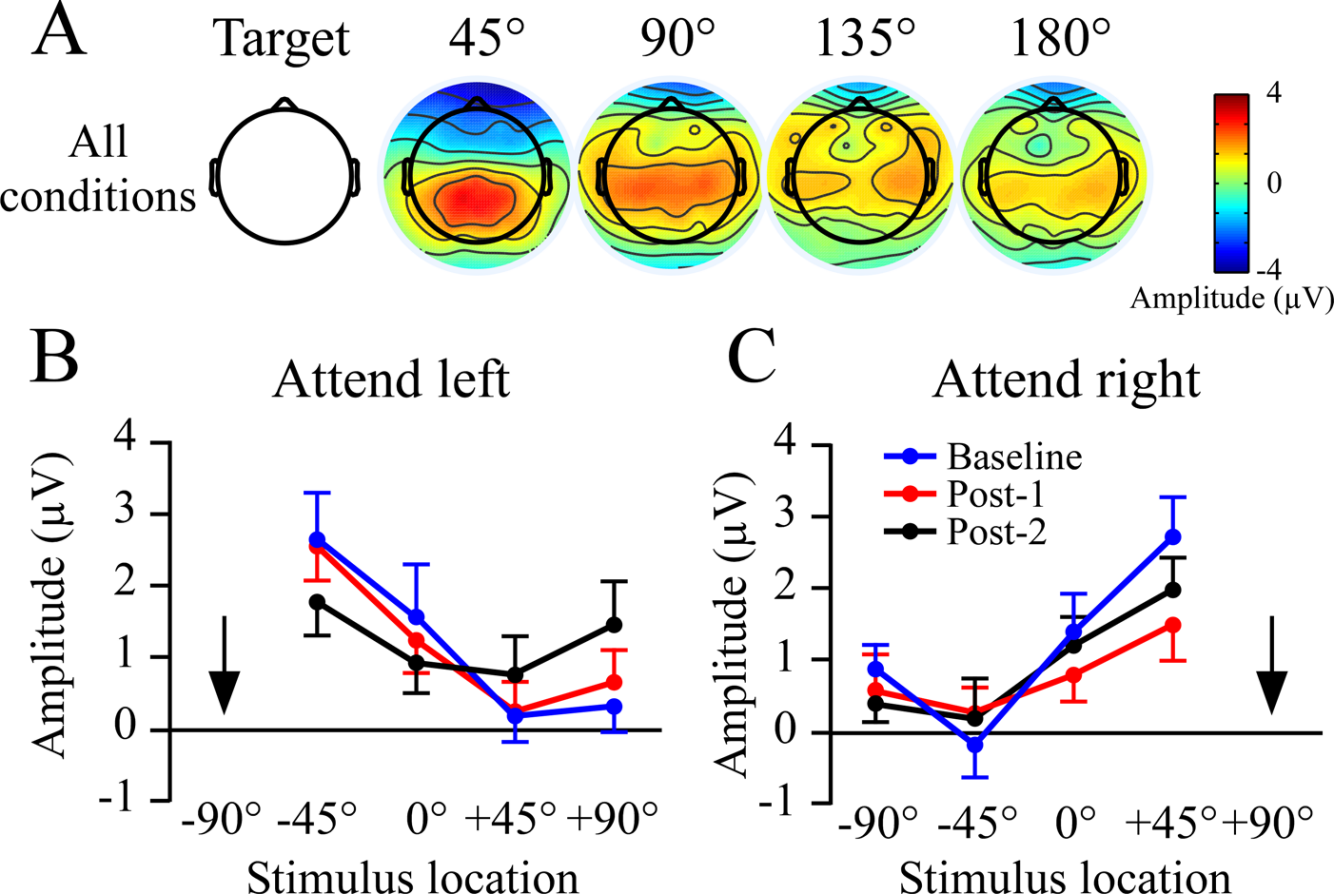
Non-target potentials between 400–460 ms at the POz site. This time window is centered on the second peak of the non-target event-related potential slope function. A) Topographic plots showing maximum positive voltage at parietal sites for the non-target nearest to the target location. There were no significant differences between attending to the left or right target, so the topographic plots are collapsed across both targets. The non-target locations are expressed relative to target location. The anterior negativity at the 45° location reflects the beginning of the frontal slow wave, which was examined in the 550–750 ms time window. B, C) Plots of potential amplitudes in the 40–460 ms time window as a function of non-target location, shown separately for the attend left and attend right conditions. The arrows indicate the attended target locations.

Results of the 400–460 ms time window analysis at POz are shown in Fig 4, which were collapsed across the three conditions. There were comparable amplitude reductions with greater distance for each target location, which plateaued at the two non-target locations farthest from the target. A 3 (condition) x 2 (target location) x 4 (non-target location) showed a robust effect of non-target location (F_3,45_ = 8.6, p < .001). There were no other significant effects or interactions, the most important of which was that rTMS did not affect ERP gradients at the 400–460 ms time window.

### 550–750 ms time window

**Fig 5:**
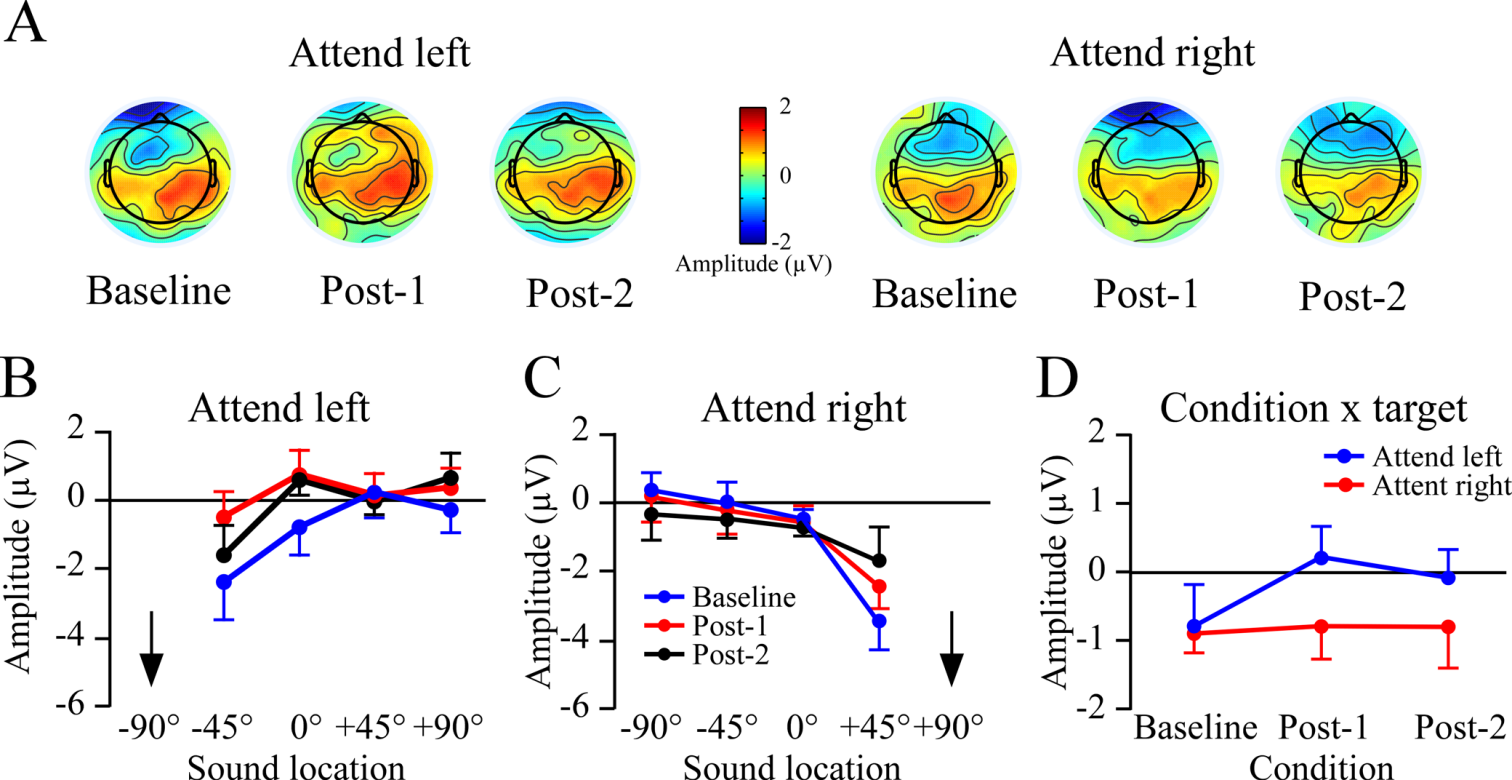
Non-target potentials between 550–750 ms at the Fz site. This time window corresponds to the third peak of non-target ERP slopes. A) Topographic plots across the Baseline, Post-1 and Post-2 rTMS conditions, shown separately for attend left and attend right targets. Results are collapsed across locations to show that when attending left there was an overall increase in positivity that which was not evident when attending to the right. B,C) Amplitude plots as a function of non-target location in the attend left and right conditions. Arrows indicate the target location.

Plots of slow wave amplitudes at Baseline, Post-1, and Post-2 blocks are shown in Fig 5 with the top row (A) showing topography collapsed across non-target locations. Window measures for each non-target location are shown in B and C. When attending to the left (-90°), overall slow wave amplitudes became more positive in Post-1, which then began to return to baseline levels in Post-2. This pattern was not evident when attending to the right target (Fig 5D). Consequently, the effect was subtle and attend-left and -right targets were examined separately. Separate 3 (condition) x 4 (non-target location) repeated measure ANOVAs were used for each target location. When attending to the left there were significant effects of condition (F_2,30_ = 3.4, p< .05) and non-target location (F_3,45_ = 3.7, p< .05). The non-target location effect indicated the slow wave potentials were most negative for the non-target sound closest to the target, and became more positive as distance increased. As with prior studies the change in amplitude across locations was less linear relative to the early time window (cf. Figs 3 and 5). A quadratic contrast across the three attend left conditions was significant (F_1,15_ = 6.7, p < .02), and reflected greater positivity regardless of location from baseline to Post-1, which then became slightly more negative in Post-2 (Fig 5B and 5D).

When the target was located at +90° there were no main effects or interactions involving condition: baseline, Post-1 and Post-2 slow wave potentials were of comparable amplitude. As when attending to the left side, there was a main effect of non-target location when attending to the right (F_3,45_ = 10.8, p < .001). The non-target location effect was due to having the most negative amplitudes near the target location, while more positive amplitudes were seen to stimuli at the other locations.

## Discussion

This study examined the impact of right inferior parietal rTMS on three ERP time windows associated with spatial attentional gradients (230–340, 400–460, 550–750 ms). At baseline all three time windows showed graded responses to non-target distractors as a function of their distance from the target location. The first and third windows were frontally located and had progressive amplitude increases (greater positivity), while the second window was maximal at posterior sites and had graded amplitude decreases (greater negativity). Slow rTMS to right inferior parietal cortex had two transient effects on the frontal measures. First, after rTMS amplitudes in the first window (230–340 ms) showed an amplitude reduction only to the most peripheral non-targets. This was present in both the attend-left and attend-right conditions, and rebounded in the second block after rTMS (between ~24–48 min after stopping rTMS). The second slope window measure was largest at parietal sites and was unaffected by rTMS. In the longest-latency window (550–750 ms) frontal slow waves became more positive when attending to the left target, regardless of sound location. Both findings were most evident in the first block after rTMS, and the measures either fully returned to baseline (230–340 ms) or showed a trend toward baseline (550–750 ms), which argues against non-specific effects such as practice or vigilance. The effects of rTMS on the 550–750 ms time window was even more specific because it was present when participants attended to the left but absent when attending to the right.

The changes in the frontal slope measures (230–340 and 550–750 ms windows) occurred in the context of progressively faster reaction times from baseline to the Post-1 and Post-2 conditions. The speeding-up of reaction times may indicate a practice effect, and it is worth considering whether practice effects may have worked against showing behavioral declines from the rTMS manipulation. The sounds were spaced far enough apart to be easily discriminable [46], whether measured using minimal audible angle [47] or directly localized using pointing [48]. Future work could examine in detail whether differences in spatial acuity (which is greatest at the midline, decreases within each hemispace, and improves at far left and right locations [43]) interact with spatial attention gradients measured with EEG. Note also that the topography and response of EEG measures as a function of distance from the attended location does not need to be reflected by a topographic arrangement of neurons coding for space at the cortical level. Indeed, single-unit recordings in mammals [49,50] and neuroimaging studies in humans [51,52] strongly support a non-topographic layout of spatial coding by auditory cortical neurons. Future work would be needed to determine the extent that the gradients measured here reflect supramodal attentional effects or are instead specific to the auditory modality. In the next section findings will be discussed with respect to the role of the right inferior parietal cortex in modulating frontal spatial attention networks.

### Gradients between 230–340 ms and the P3a component

The robust, linear increase in frontal amplitudes with greater distractor-to-target distance for the first window measure replicated previous findings in this task [14]. The window measure covers the latency range of the P3a ERP component, which is associated with attention capture by infrequent, task-irrelevant stimuli [15,53]. The acoustic P3a is generated by a distributed cortical network including the prefrontal, parietal, and auditory cortex [54,55]. The transient amplitude decreases at the most peripheral locations after rTMS were symmetrical regardless of attended location, which contrasts with the asymmetrical stimulation of the right inferior parietal cortex. Neuroimaging studies suggest that a ventral attention network in the right hemisphere is particularly engaged when orienting to distractors that relate to the subject’s task [56]. The idea that the right parietal cortex orients attention to both the left and right sides was originally an inference based on lesion data from hemineglect patients [19,57], and was supported by our previous study using independent component analysis in healthy adults [3]. There is also structural MRI evidence for a right hemisphere asymmetry in white matter connectivity between posterior temporal/parietal areas and the superior parietal lobe [58], which may have been affected by the rTMS.

Examination of the ERPs across the four non-target locations suggested that the latency of the P3a peak within the 230–340 ms window decreased as non-targets were presented farther from the target location. For this reason we also analyzed the peak latency of the P3a. P300 peaks are traditionally related to stimulus classification, although this idea has been developed around the P3b which is elicited by targets requiring a response [59–61]. Additional work would be needed to determine if the focused attention at the target location impeded stimulus classification for nearby non-target stimuli. The P3a latency results are inconsistent with analog, or “spotlight”, shifts of spatial attention because the P3a latency decreases with greater distance from the target. Analog attention shifts would instead have increasing latencies as non-targets are presented farther from the target location. We also note that evidence from visual attention studies generally does not support the idea of analog attention shifts [62].

### 400–460 ms window

The second of the three ERP gradients developed between ~400–460 ms, and had a negative slope centered over parietal sites. Unlike the gradients in the first and third time windows, rTMS had no significant effects on this gradient; neither the form nor overall voltage levels were influenced by rTMS. The lack of any notable effects further illustrates the selectivity of the rTMS manipulation. This second gradient time-period was only recently reported [3], and its functional properties have not been thoroughly investigated. Future work may target dorsal parietal cortex to determine if rTMS interferes more directly with possible generators of this gradient.

It is worth noting that the maximum slope was bilaterally centered above dorsal parietal areas, and was near, but not at, the site of rTMS delivered to right ventral parietal cortex. The dorsal and ventral parietal cortices are association regions with a panoply of structural and functional differences. For example, the inferior parietal cortex has recently been divided up into seven regions using objective cytoarchitectonic methods [63], which are associated with differences in anatomical connectivity [64]. Thus additional work will be needed to clearly define the impact of TMS pulses directed to the inferior parietal cortex on cortical function. This goal is especially elusive in the present study because the TMS site was defined by using a standardized location based on scalp coordinates rather than neural structure or functional activation.

### 550–750 ms window and slow waves

In traditional auditory oddball paradigms slow waves in the 550–750 ms time range are modulated by task demands and other experimental variables [65–71]. Here, the frontal slow waves were elicited by non-target distractors, and were not associated with motor responses because subjects did not press the button to the non-targets that were analyzed. The frontal slow waves showed that a persistent record of a distractor’s location relative to the target is maintained well after the time when responses could be made to targets (on average ~500 ms). The functional properties of slow wave gradients on this task have not been tested, but long-lasting slow waves may have a role in attention control between trials, for example sequence effects [72].

As in previous reports [3,14], the frontal slow wave gradients were more focal relative to the earlier time windows that had clear linear gradients. The relative spatial coding of non-target locations, that is the slope, was not affected by rTMS. This suggests that the right parietal rTMS had a modulatory effect on the slow waves but did not disrupt the spatial coding of the slow wave signal. The effects of rTMS were observed only when attending to the left side, which provides an internal control for any non-specific effects of rTMS. The specificity of rTMS effects to left side attention task bears a similarity to patients with hemineglect, who usually neglect only the left side of space [17,18,22,24,73]. However, an important limitation is that rTMS to the left inferior parietal cortex was not tested. Consequently, it is not known if parietal rTMS in this task would mimic the effects of lesions, with little effect of left rTMS, or instead would induce a symmetrical effect with left rTMS inducing more positive slow waves when attending to the right target.

### Relevance to cortical attention networks

We speculate that the gradient-like responses observed in the three time windows may reflect activities within the dorsal attention system identified in neuroimaging studies [56]. The main similarities are that the present task assesses voluntary attention, which is expected to engage the dorsal attention system, and the basic profile of gradient responses when attending to the left and right were remarkably symmetrical. However, recent behavioral evidence suggests that the interaction between the dorsal and ventral frontal-parietal networks is key to efficient allocation and shifting of spatial attention [74]. Non-spatial attributes, such as reorienting of attention, arousal, and target detection recruit the right lateralized ventral attention system, including the temporoparietal junction, inferior parietal lobule, superior temporal gyrus, and inferior frontal gyrus [26,75]. The impact of rTMS to the right inferior parietal cortex may indirectly affect dorsal network function, as has been suggested to occur in hemineglect [26,75]. Lastly, both the first and third time windows were centered over frontal areas, which suggests rTMS effects propagated through neural networks to have an effect on distant frontal structures.

## Supporting Information

S1 Table Mean values and standard errors of the three window measures and P3a latencies of the four non-target locations across conditions and target locations. (TIF)
