# Supplementary figures and images for: The Role of Right Inferior Parietal Cortex in Auditory Spatial Attention: A Repetitive Transcranial Magnetic Stimulation Study
Source: PLoS One. 2015 Dec 4;10(12):e0144221. doi: 10.1371/journal.pone.0144221 (PMC4670170; doi:10.1371/journal.pone.0144221)

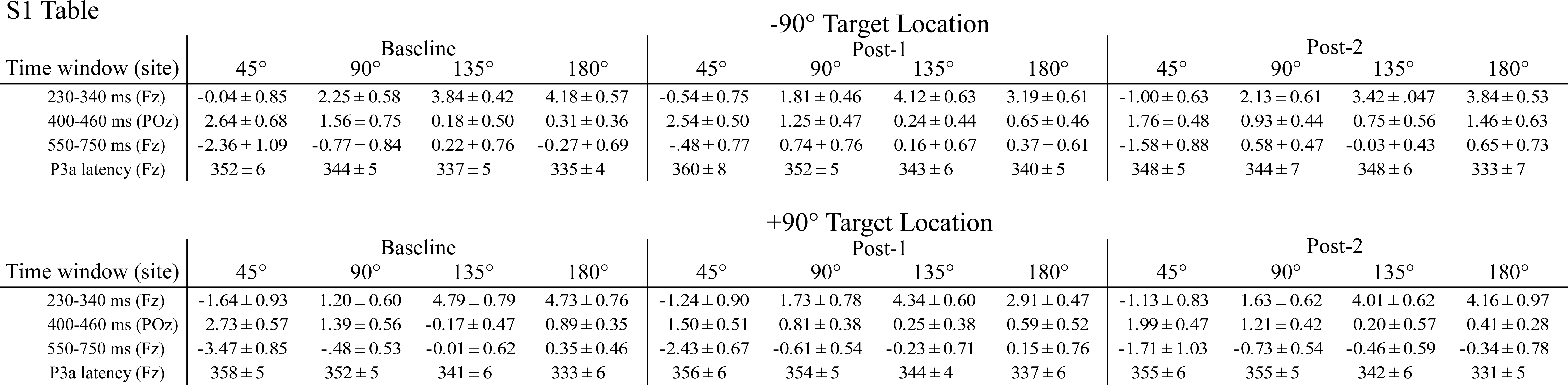

Supplement: S1 Table — (TIF) [file pone.0144221.s001.tif]
